# Cut-off values of latent infection in patients with rapid migration following bipolar hip hemiarthroplasty

**DOI:** 10.1186/s12891-016-0876-3

**Published:** 2016-01-19

**Authors:** Takao Setoguchi, Hirotaka Kawakami, Yasuhiro Ishidou, Hideki Kawamura, Junichiro Nishi, Takako Yoshioka, Hironori Kakoi, Satoshi Nagano, Masahiro Yokouchi, Akihide Tanimoto, Setsuro Komiya

**Affiliations:** The Near-Future Locomotor Organ Medicine Creation Course (Kusunoki Kai), Graduate School of Medical and Dental Sciences, Kagoshima University, 8-35-1 Sakuragaoka, Kagoshima, 890-8520 Japan; Department of Orthopaedic Surgery, Graduate School of Medical and Dental Sciences, Kagoshima University, Kagoshima, Japan; Department of Medical Joint Materials, Graduate School of Medical and Dental Sciences, Kagoshima University, Kagoshima, Japan; Kagoshima University Hospital, Infection Control Team, Kagoshima, Japan; Department of Microbiology, Graduate School of Medical and Dental Sciences, Kagoshima University, Kagoshima, Japan; Department of Molecular and Cellular Pathology, Graduate School of Medical and Dental Sciences, Kagoshima University, Kagoshima, Japan

**Keywords:** Bipolar hip hemiarthroplasty (BHA), Migration, Radiographic evaluation, Periprosthetic joint infection, Cut-off value prosthesis survival, Polymorphonuclear leukocyte (PMN)

## Abstract

**Background:**

Although most patients achieve favorable results following bipolar hip hemiarthroplasty (BHA), some experience rapid migration of the prosthesis. We retrospectively reviewed 18 patients with BHA that necessitated revision.

**Methods:**

We examined soft tissues obtained from periprosthetic lesions. In total, 18 patients with pain and acetabular migration of the BHA prosthesis were included. The patients were divided into a polymorphonuclear leukocyte (PMN)-positive (≥5 PMNs per high-power field [HPF]) and PMN-negative (<5 PMNs/HPF) group.

**Results:**

Pathological findings showed that 11 patients were PMN-positive, which was indicative of infection. All patients in the PMN-positive group showed no polyethylene particles or foreign body giant cells, while all patients in the PMN-negative group showed polyethylene debris or foreign body giant cells (*p* < 0.001). BHA survival, C-reactive protein (CRP) levels, and the Japanese Orthopaedic Association (JOA) hip score were significantly different between the PMN-positive and PMN-negative group (*p* < 0.01). A BHA survival cut-off value of 3270 days was diagnostic for PMN positivity (sensitivity: 100 %; specificity: 100 %). The cut-off values for CRP and the JOA hip score were 0.43 mg/dl and 56 points, respectively. Four of 11 PMN-positive patients showed no clinical symptoms of infection (asymptomatic PMN-positive group). BHA survival, CRP levels, and JOA hip scores were significantly different between the asymptomatic PMN-positive and PMN-negative group (*p* < 0.05). A BHA survival cut-off of 3270 days was diagnostic for asymptomatic PMN positivity (sensitivity: 100 %; specificity: 100 %). The cut-off values for CRP and the JOA hip score were 0.43 mg/dl and 57 points, respectively.

**Conclusion:**

Our findings suggest that some portion of rapid BHA prosthesis migration is caused by mild infection. Careful pathological examination should be performed to identify infection before removal of the BHA prosthesis in patients who develop migration within 9 years.

## Background

Bipolar hip hemiarthroplasty (BHA) is currently the gold standard treatment for unstable femoral neck fractures and was introduced by Bateman in 1974 [1]. BHA was developed to reduce both wear on the acetabular cartilage and acetabular migration, both of which frequently occurred with unipolar prostheses of the Moore and Thompson type [2–4]. Although BHA has achieved moderately successful results, revision surgery is needed when the BHA prosthesis migrates [5–8].

Because the clinical symptoms of periprosthetic joint infection (PJI) are not always reliable, diagnosis relies on a combination of blood tests, culture, and histological examination [9]. It has been reported that accumulation of polymorphonuclear leukocytes (PMNs) in periprosthetic tissue is highly reliable evidence for PJI [10]. We performed histopathological examinations and found ≥5 PMNs per high-power field (HPF) in the periprosthetic tissues from 11 of 18 patients who underwent BHA revisions due to acetabular migration of the prosthesis. The patients were divided into a PMN-positive and PMN-negative group. We examined which factors were associated with PMN positivity.

## Methods

### Patients

This study was based on the data of 18 patients who had been hospitalized in the Department of Orthopaedic Surgery of Kagoshima University Hospital from April 2006 to January 2014. All patients had hip joint pain and acetabular migration of the BHA prosthesis. The mean age of the 11 women and seven men was 65 years (range, 37–87 years). The primary diagnosis was idiopathic osteonecrosis of the femoral head in six patients and fracture of the femoral neck in 12. The median survival of the BHA was 2830 days (range, 56–9291). A preoperative diagnosis of infection was made based on clinical presentation, laboratory data, and diagnostic imaging. The clinical symptoms of infection were defined as pyrexia, local swelling and heat, tenderness, and the presence of pus before the time of BHA prosthesis removal [11, 12]. Laboratory evidence of infection was an elevated C-reactive protein (CRP) level of >1 mg/dl. Of the 18 patients, four had clinical signs of local infection with an accompanying fistula and purulent discharge. Another three patients showed a CRP level of >1 mg/dl. Eight of the 18 patients were preoperatively diagnosed with aseptic loosening. The remaining 10 patients were preoperatively diagnosed with PJI. One to three periprosthetic tissue specimens of each patient were examined postoperatively by histopathological examination. Several articles have reported that 5 PMNs/HPF is a suitable diagnostic threshold for diagnosis of PJI [10, 13]. The patients were divided into a PMN-positive (≥5 PMNs/HPF) and PMN-negative (<5 PMNs/HPF) group. In addition, the PMN-positive group was subdivided into a symptomatic PMN-positive group and an asymptomatic PMN-positive group (no pus, pyrexia, local swelling, heat, or tenderness and a CRP level of ≤1 mg/dl).

### Statistical analysis

The distributions of the variables of each group were assessed using the Kolmogorov–Smirnov test. Significant differences (*p* < 0.05) between groups were determined using Student’s *t*-test, the Mann–Whitney *U* test, and Fisher’s exact test. The BHA survival rate was calculated using the Kaplan–Meier method and the log-rank test. Receiver operating characteristic (ROC) curve analysis was performed to determine the cut-off value with maximum sensitivity and specificity. All statistical analyses were performed using Excel Statistics 2012 (SSRI, Osaka, Japan).

### Pathological examination

Specimens for histopathological examination were obtained from one to three sites of the periprosthetic tissues. The specimens were stained with hematoxylin and eosin. Multiple sections from each site were examined for the number of PMNs per HPF (×400) and presence of polyethylene particles or foreign body giant cells in more than 10 separate microscopic fields. All histopathological examinations were performed by a skilled pathologist with no knowledge of the data. Several articles have reported that 5 PMNs/HPF is a suitable diagnostic threshold for diagnosis of PJI [9, 14]; therefore, we regarded 5 PMNs/HPF as positive for infection.

### Ethics statement

This research protocol was approved by the Ethics Committee on Clinical Research at Kagoshima University Hospital (Pathological examination of periprosthetic infection for rapidly migrating hip hemiarthroplasty: No. 437).

### Consent statement

All patients gave their informed written consent for participation in this clinical study.

## Results

### Invasion of periprosthetic tissues by PMNs in 11 of 18 patients

The patients were divided into a PMN-negative (<5 PMNs/HPF) (*n* = 7) and PMN-positive (≥5 PMNs/HPF) (*n* = 11) group. The demographic data are summarized in Table 1. All patients in the PMN-positive group showed no polyethylene particles or foreign body giant cells, while all patients in the PMN-negative group showed polyethylene debris or foreign body giant cells (*p* < 0.001) (Table 2). These findings suggest that polyethylene particles caused aseptic migration of the BHA prosthesis in the PMN-negative groups and that migration of the BHA prosthesis in the PMN-positive group was caused by infection.

**Table 1:**
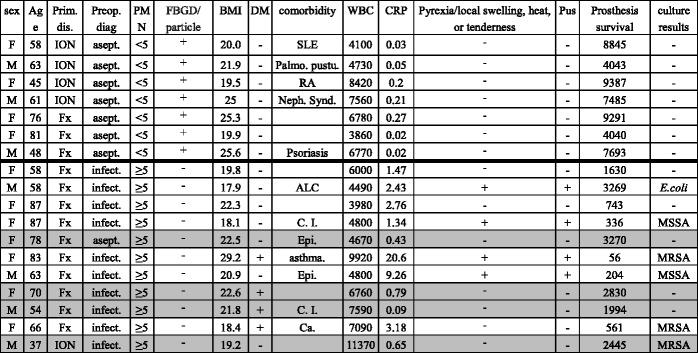
Clinicopathological data of the patients

Grey lines show asymptomatic PMN-positive groups

*F* Female, *M* Male, *Fx* Fracture, *ION* Idiopathic osteonecrosis of the femoral head, *Prim. dis.* Primary disease, *Preop. diag.* Preoperative diagnosis, *asept.* aseptic loosening, *infect.* infection, *FBGD* foreign body giant cell, *particle* particles, *BMI* body mass index, *DM* Diabetes mellitus, *Palmo. pustu.* Palmoplantar pustulosis, *RA* Rheumatoid arthritis, *Neph. Synd.* Nephrotic syndrome, *ALC* Alcoholic liver cirrhosis, *C. I.* Cerebral infarction, *Epi.* Epilepsy, *athma.* Bronchial asthma, *Ca.* Multiple cancer, *WBC* white blood cells

**Table 2:** Clinicopathological data in which the differences between PMN-negative and PMN-positive patients

Polymorphonuclear leukocytes	PMN-negative	PMN-positive	*p*-value
Presence of polyethylene debris or foreign body giant cell	7/7	0/11	<0.001
Age (years, mean ± SD)	61.7 ± 13.3	67.4 ± 15.6	=0.779
Female (%)	0.57	0.64	=0.783
Body Mass Index (median)	21.9	20.9	=0.258
Diabetes mellitus	0/7	4/11	=0.137
Primary disease (ION:fracture)	4:3	1:10	=0.052
W.B.C. (cells/mL, mean ± SD)	6031 ± 1793	6497 ± 2382	=0.664

### Significant differences in BHA survival, CRP level, and Japanese Orthopaedic Association hip score between PMN-positive and PMN-negative groups

**Fig. 1:**
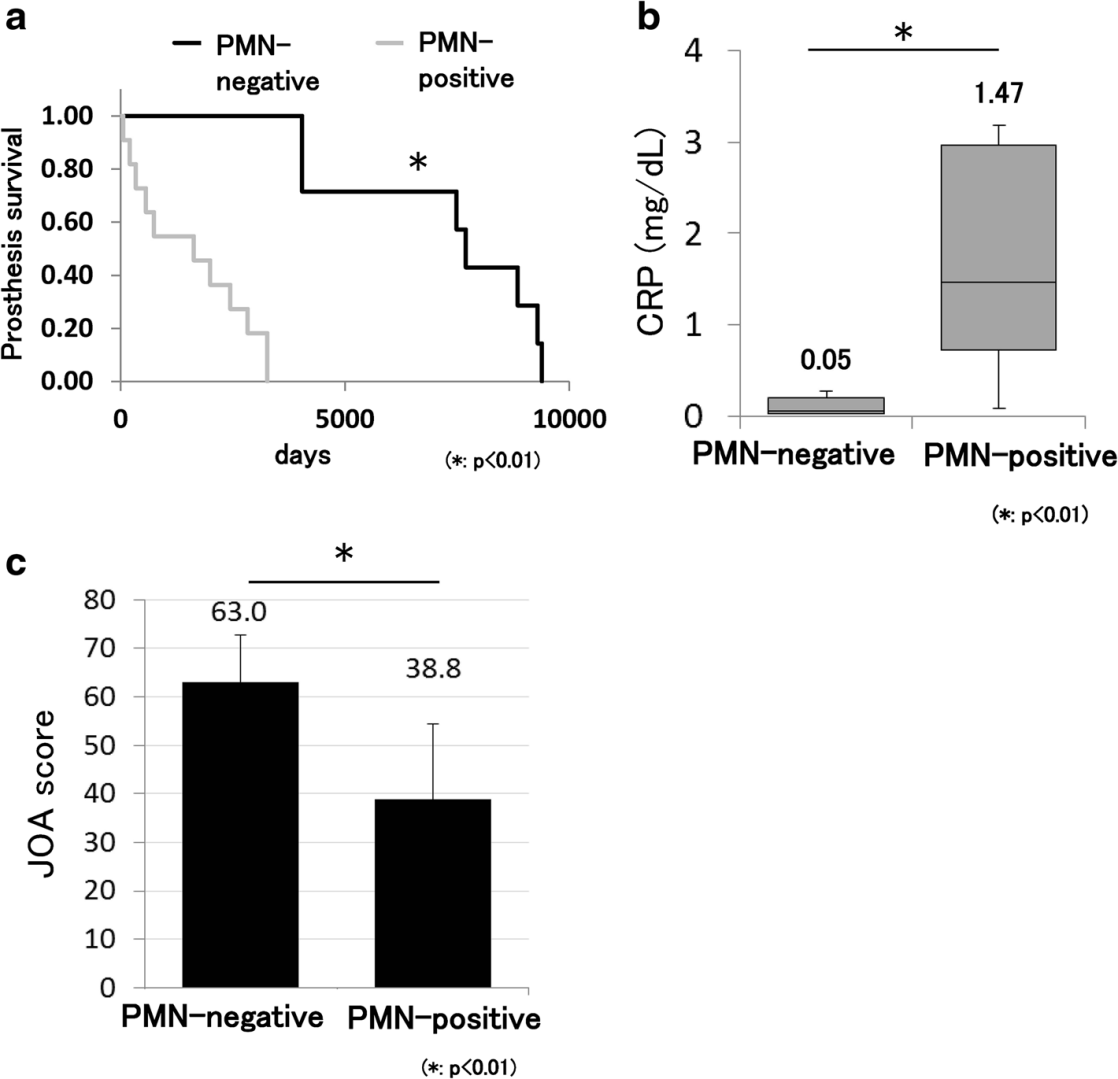
Significant differences between PMN-positive and PMN-negative groups. **a** Kaplan–Meier analysis showed that BHA survival was significantly shorter in the PMN-positive than in the PMN-negative group. **b** CRP levels were significantly different between the PMN-positive and PMN-negative groups (*p* < 0.01). **c** JOA hip scores were significantly different between the PMN-positive and PMN-negative groups (*p* < 0.01)

**Fig. 2:**
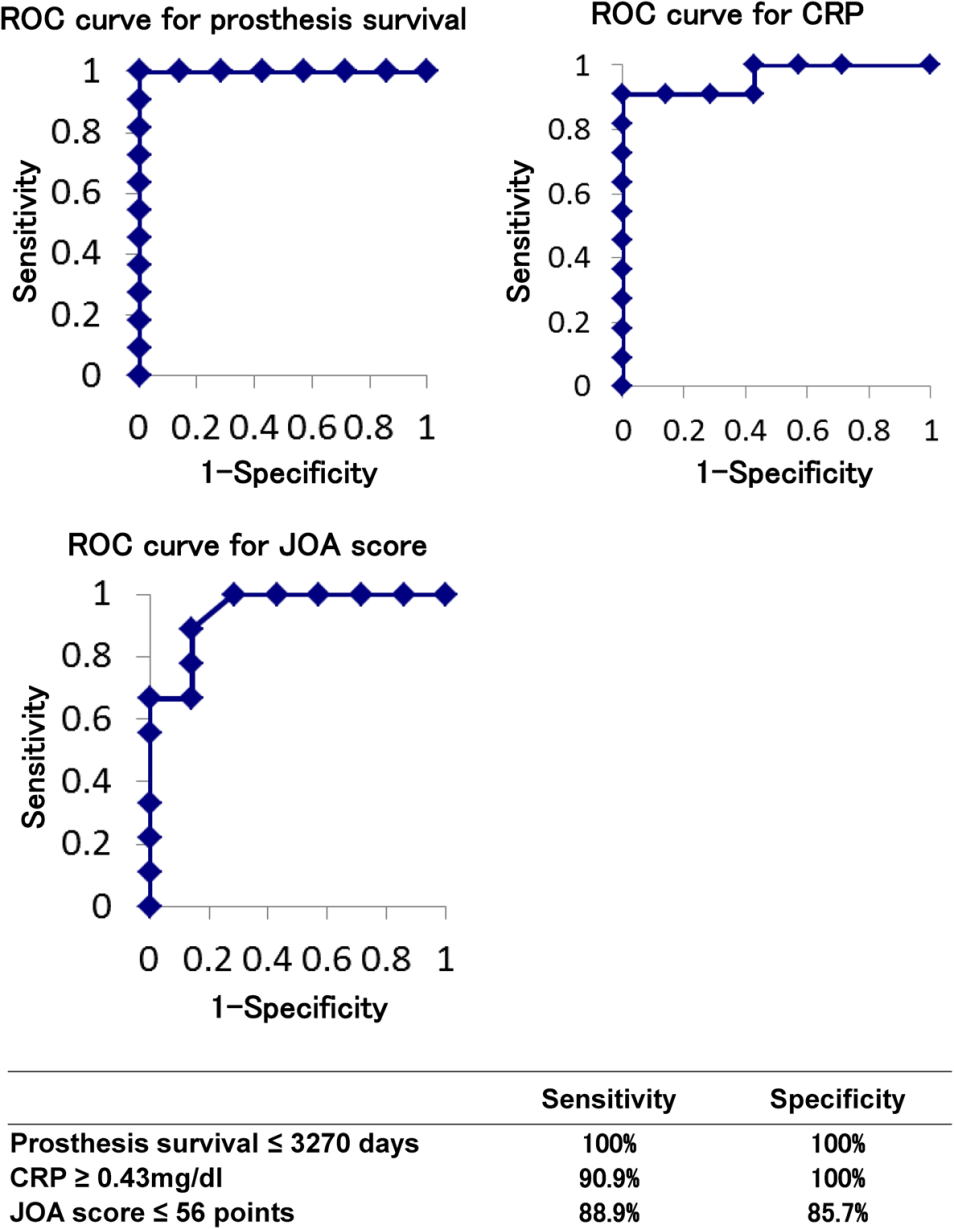
Diagnostic criteria for PMN positivity

Both the Kaplan–Meier and log-rank tests showed a statistically significant difference in BHA survival between the PMN-positive and PMN-negative groups (*p* < 0.01) (Fig. 1a). The median level of CRP just prior to BHA removal showed a statistically significant difference between the PMN-positive and PMN-negative groups (*p* < 0.01) (Fig. 1b). In addition, the total average clinical Japanese Orthopaedic Association (JOA) hip score just before BHA removal showed a statistically significant difference between the PMN-positive and PMN-negative groups (*p* < 0.01) (Fig. 1c). Pain in the hip joint resulted in a lower JOA hip score in the PMN-positive group. The other factors were not significantly different (Table 2). The ROC curve analysis showed that the cut-off values of BHA survival, CRP level, and JOA hip score that were diagnostic for PMN positivity were 3270 days (sensitivity: 100.0 %; specificity: 100.0 %), 0.43 mg/dl (sensitivity: 90.9 %; specificity: 100.0 %), and 56 points (sensitivity: 88.9 %; specificity: 85.7 %), respectively (Fig. 2).

### Significant differences in BHA survival, CRP level, and JOA hip score between asymptomatic PMN-positive and PMN-negative groups

**Fig. 3:**
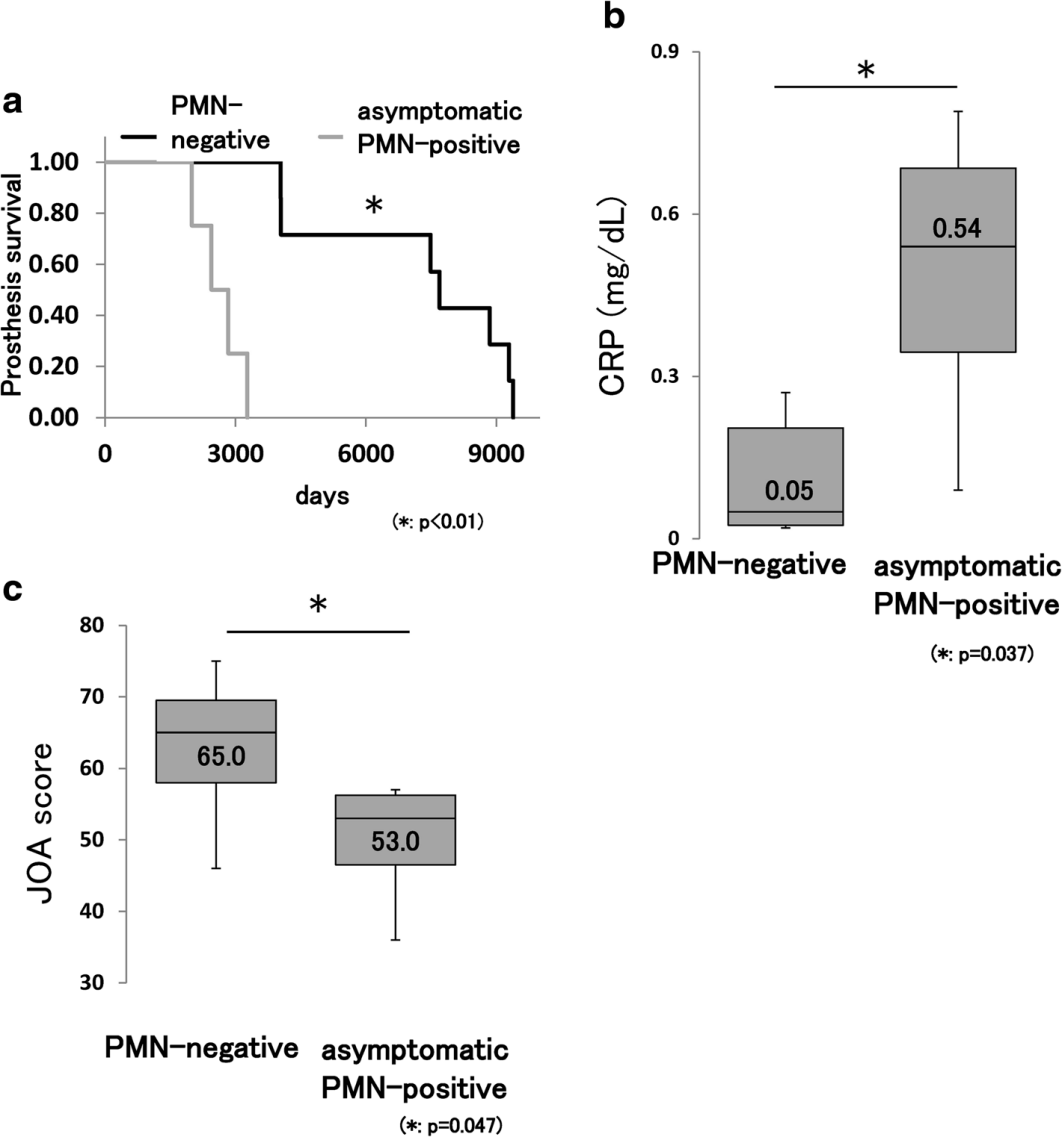
Significant differences between asymptomatic PMN-positive and PMN-negative groups. Asymptomatic PMN positivity was defined as a CRP level of <1 mg/dl and no local swelling, local heat, tenderness, or evidence of pus. **a** Kaplan–Meier analysis showed that BHA survival was significantly shorter in the asymptomatic PMN-positive than in the PMN-negative group. **b** CRP levels were significantly different between the asymptomatic PMN-positive and PMN-negative groups (*p* < 0.05). **c** JOA hip scores were significantly different between the asymptomatic PMN-positive and PMN-negative groups (*p* < 0.05)

**Fig. 4:**
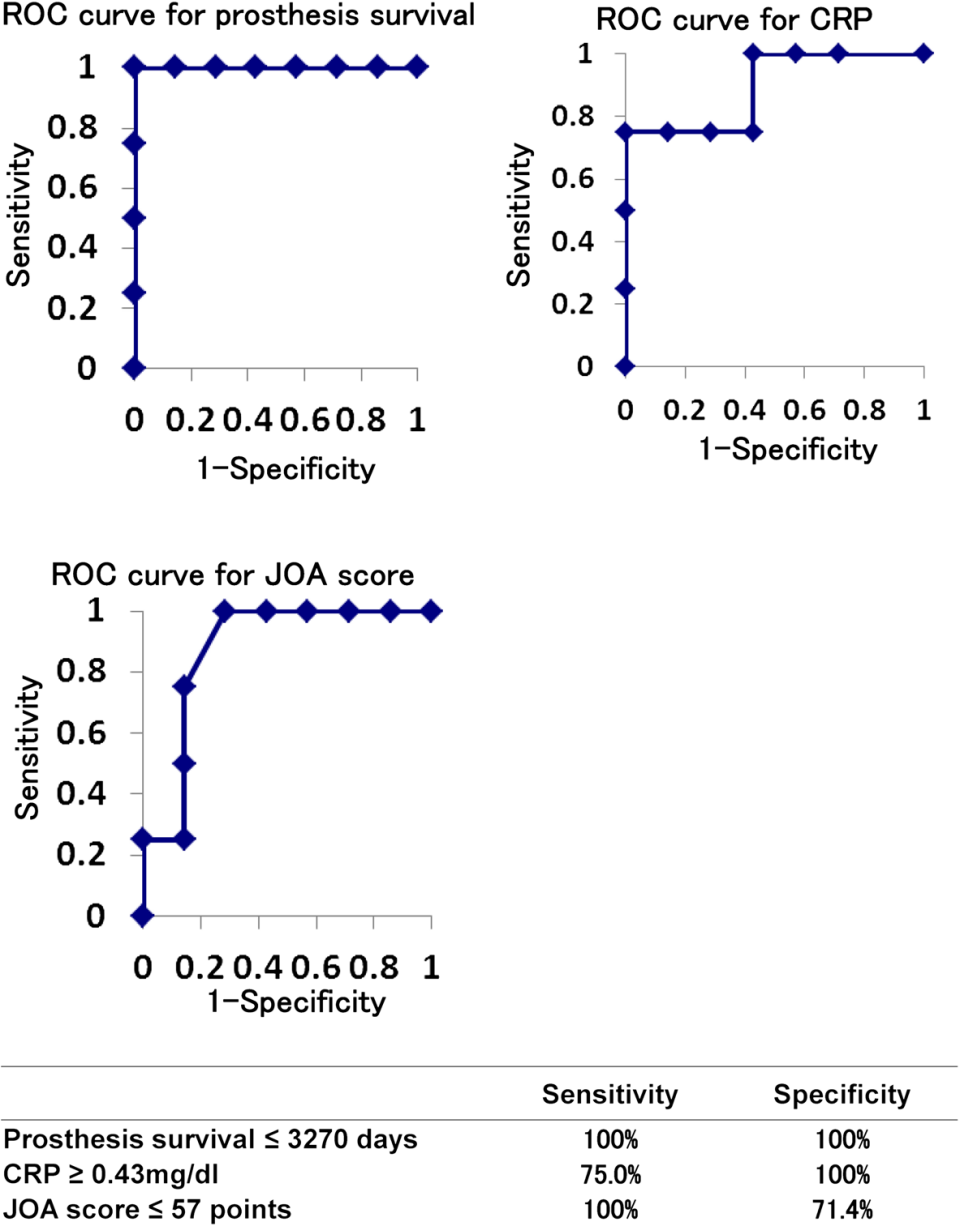
Diagnostic criteria for asymptomatic PMN positivity

Four patients in the PMN-positive group showed no clinical symptoms of infection (asymptomatic PMN-positive group: gray lane in Table 1). Both the Kaplan–Meier and log-rank test showed a statistical difference in BHA survival (*p* < 0.01) (Fig. 3a). The median level of CRP just prior to BHA removal showed a statistically significant difference between the asymptomatic PMN-positive and PMN-negative groups (*p* = 0.037) (Fig. 3b). In addition, the median JOA hip score just before BHA removal showed a statistically significant difference (*p* = 0.047) (Fig. 3c). The other factors were not significantly different (Table 3). The ROC curve analysis showed that the cut-off values of BHA survival, CRP level, and JOA score that were diagnostic for asymptomatic PMN positivity were 3270 days (sensitivity: 100.0 %; specificity: 100.0 %), 0.43 mg/dl (sensitivity: 75.0 %; specificity: 100.0 %), and 57 points (sensitivity: 100.0 %; specificity: 71.4 %), respectively (Fig. 4).

**Table 3:** Clinicopathological data in which the differences between PMN-negative and asymptomatic PMN-positive patients

Polymorphonuclear leukocytes	PMN negative	Asymptomatic PMN-positive	*p*-value
Presence of polyethylene debris or foreign body giant cell	7/7	0/4	=0.003
Age (years, median)	61.0	62.0	=0.850
Female (%)	0.57	0.50	=0.652
Body Mass Index (median)	21.9	22.1	=0.571
Diabetes mellitus	0/7	2/4	=0.109
Primary disease (ION:fracture)	4:3	1:3	=0.359
W.B.C. (cells/mL, median)	6770	7175	=0.450

## Discussion

According to the guidelines on prosthetic joint infection of the Infectious Diseases Society of America, the accumulation of PMNs in periprosthetic tissue is highly suggestive of PJI [13]. In our study, 11 patients experienced migration of the BHA prosthesis within 9 years and showed accumulation of PMNs in the periprosthetic tissue, consistent with the PJI criteria. The ROC analysis showed that the cut-off value for BHA survival between the PMN-positive and PMN-negative groups was 3270 days (sensitivity: 100 %; specificity: 100 %). Although the sample size was small, these highly specific findings suggest that migration of the BHA prosthesis within 9 years might be caused by PJI.

The long-term results of BHA are generally satisfactory [15–18]. The acetabular migration distance following BHA is reportedly 0.5–3.5 mm at 5–15 years postoperatively [5]. Kim et al. [19] reported that the mean degeneration rate of acetabular cartilage was 0.34 ± 0.35 mm/year. However, Nakata et al. [20] reported progressive migration and massive acetabular osteolysis at ≥5 years after BHA. Several groups have reported that revision THA is required because of early acetabular migration [18, 21]. Why some patients develop rapid migration of the BHA prosthesis is unclear. Our findings suggest the involvement of infection, although the patients showed no clinical symptoms of infection. In our PMN-positive group (*n* = 11), four patients had a CRP level of <1 mg/dl and no local swelling, local heat, tenderness, or evidence of pus. Our findings suggest that if the BHA prosthesis migrates within 9 years, diagnosis should rely on pathological examination of the periprosthetic tissues, even when patients show no clinical symptoms of infection.

Other explanations of rapid migration following BHA have been proposed. Several groups reported that excess outer-bearing motion in the absence of inner-bearing motion leads to a high degree of wear particles and thus osteolysis and migration [20, 22–24]. However, wear debris as a cause of rapid migration was unlikely in our patients because the pathological findings showed no evidence of polyethylene debris or foreign body reactions in any of the 11 PMN-positive patients, while all of the PMN-negative patients showed polyethylene debris or foreign body giant cells. These findings reinforce the idea that early migration of the BHA within 9 years was caused by infection in this study.

## Conclusions

Although most patients who undergo BHA have satisfactory results, a small number of patients experience rapid migration and failure of the BHA prosthesis. Our findings suggest that if the BHA prosthesis migrates within 9 years, careful inspection including pathological examination should be performed before revision to prevent sustained infection of the revised THA.

## Abbreviations

BHA: Bipolar hip hemiarthroplasty
PJI: Periprosthetic joint infection
PMNs: Polymorphonuclear leukocytes
CRP: C-reactive protein
JOA: Japanese Orthopaedic Association
HPF: High-power field
ROC: Receiver operating characteristic
THA: Total hip arthroplasty
